# A Synthetic Thiourea-Based Tripodal Receptor that Impairs the Function of Human First Trimester Cytotrophoblast Cells

**DOI:** 10.3390/ijerph110707456

**Published:** 2014-07-21

**Authors:** Darijana Horvat, Maryam Emami Khansari, Avijit Pramanik, Madhava R. Beeram, Thomas J. Kuehl, Md. Alamgir Hossain, Mohammad Nasir Uddin

**Affiliations:** 1 Department of Obstetrics & Gynecology, Texas A&M Health Science Center College of Medicine/Scott & White Hospital, Temple, TX 76508, USA; E-Mails: DHORVAT@sw.org (D.H.); TKUEHL@sw.org (T.J.K.); 2 Department of Chemistry and Biochemistry, Jackson State University, Jackson, MS 39217, USA; E-Mails: maryam.emami_khansari@students.jsums.edu (M.E.K.); avijit.pramanik@jsums.edu (A.P.); alamgir.hossain@jsums.edu (M.A.H.); 3 Department of Pediatrics, Texas A&M Health Science Center College of Medicine/Scott & White Hospital, Temple, TX 76508, USA; E-Mail: mbeeram@sw.org

**Keywords:** angiogenic, cardiotonic steroids, cell signaling, cytotrophoblast, preeclampsia, thiourea

## Abstract

A synthetic tripodal-based thiourea receptor (PNTTU) was used to explore the receptor/ligand binding affinity using CTB cells. The human extravillous CTB cells (Sw.71) used in this study were derived from first trimester chorionic villus tissue. The cell proliferation, migration and angiogenic factors were evaluated in PNTTU-treated CTB cells. The PNTTU inhibited the CTBs proliferation and migration. The soluble fms-like tyrosine kinase-1 (sFlt-1) secretion was increased while vascular endothelial growth factor (VEGF) was decreased in the culture media of CTB cells treated with ≥1 nM PNTTU. The angiotensin II receptor type 2 (AT_2_) expression was significantly upregulated in ≥1 nM PNTTU-treated CTB cells in compared to basal; however, the angiotensin II receptor, type 1 (AT_1_) and vascular endothelial growth factor receptor 1 (VEGFR-1) expression was downregulated. The anti-proliferative and anti-angiogenic effect of this compound on CTB cells are similar to the effect of CTSs. The receptor/ligand affinity of PNTTU on CTBs provides us the clue to design a potent inhibitor to prevent the CTS-induced impairment of CTB cells.

## 1. Introduction

Preeclampsia (preE) is a hypertensive disorder unique to pregnancy with multiple etiologies that affects about 3 to 10 percent of pregnant women [1,2]. In a rat model of preE, it has been shown that urinary marinobufagenin (MBG) levels are elevated prior to the development of hypertension indicating that it may play a key role in the pathogenesis of preE [3]. Cardiotonic steroids (CTSs), such as MBG, cinobufotalin (CINO), and ouabain (OUB), are endogenous inhibitors of Na^+^/K^+^ ATPase [4,5,6,7]. It has been demonstrated that CTSs impair cytotrophoblast (CTB) cell function [8,9]. The receptor/receptors of CTSs on CTBs has/have not been fully understood. We have studied the effect of CTSs on pregnancy using CTB cells in order to determine their role in preE [8,9]. The CTSs inhibited CTB proliferation, migration, invasion, and ERK1/2 phosphorylation and they activated Jnk1/2 phosphorylation, p38 phosphorylation, and apoptosis evaluated by caspase 3/7 and annexin-five staining [8,9]. The CTSs also arrested cell cycle progression without causing a cytotoxic effect on the cells [8,9]. We have demonstrated that CTS induced impairment of CTBs and endothelial cells function occurs via the modulation of MAPK signaling, cell cycle arrest, and the activation of apoptosis [10,11,12]. 

**Figure 1:**
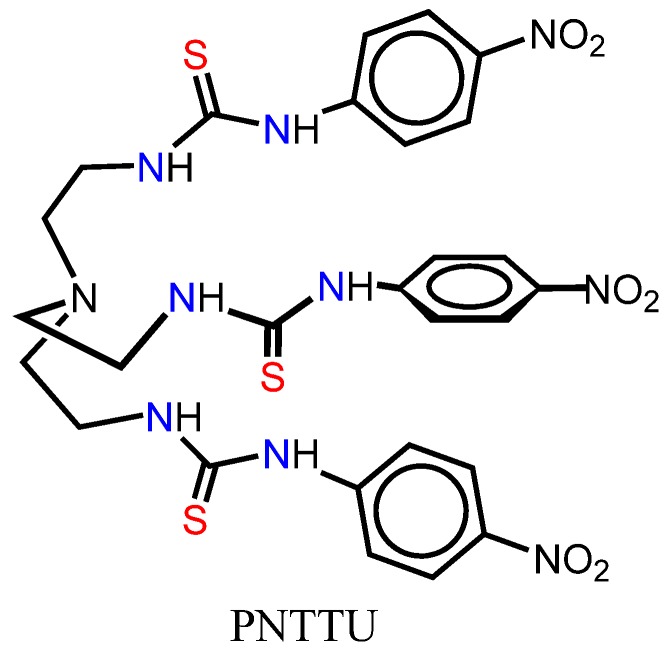
Chemical structure of PNTTU (*para-*nitro tripodal thiourea)

Urea- or thiourea-based synthetic receptors are known to interact with an anion under neutral conditions [13,14,15,16]. Recently, it has been reported that such compounds have the potential to act as anticancer agents through transmembrane transport mechanisms of anions *in vitro* [17]. In this study, we have been interested in using a thiourea-based molecule, PNTTU (*para-*nitro tripodal thiourea, Figure 1) as a cell surface receptor for CTSs. The basic feature of PNTTU is that it contains three functional groups as active thiourea (HN(C=S)NH) groups in the attached three arms forming a triopodal cavity which was previously shown to bind anions [18,19,20]. The presence of three *p*-nitro groups as electron withdrawing substituents enhances the acidity of the attached NH groups, thereby increasing the overall activity of the ligand. This hypothesis was supported by a calculation of the electrostatic potential surfaces of a related compound at the M06-2X/6-31G(d,p) level of theory, showing the most positive potential on the NH groups [21]. In addition, the conformational flexibility with six H-donor groups may allow the ligand to interact with active sites of a cell. 

CTSs have a specific binding site on the extracellular loops (TM1–TM2, TM5–TM6, and TM7–TM8) of the α subunit of Na^+^/K^+^ ATPase [22]. The sensitivity of the sodium pump to CTSs is controlled by multiple mechanisms in addition to the tissue specificity of α and β isoform distributions [22]. CTSs are stimulated to promote natriuresis via inhibition of the Na^+^/K^+^ pump in renal tubules and are likely to exhibit a prohypertensive action via inhibition of the Na^+^/K^+^ pump in vascular sarcolemma [4,23]. Recently, several research teams proposed that CTSs are also involved in Na^+^-independent signaling. This hypothesis was based on data showing that at lower concentrations CTSs augment cell proliferation [24,25], DNA synthesis [24], mitogen-activated protein kinase activity [5,24,26], and the production of reactive oxygen species [6,27] without significant inhibition of the Na^+^/K^+^ pump and elevation of [Na^+^]i. Another possible approach to interfere with the effect of CTSs is the inhibition of the Na^+^/Ca^2+^ exchanger, NCX1 [27]. CTSs inhibit Na^+^/K^+^ ATPase in vascular smooth muscle cells; the elevation of local Na^+^ facilitates Ca^2+^ entry through NCX1, resulting in vasoconstriction [7]. There are two types of CTSs, cardenolides (OUB) and bufadienolides (MBG and CINO). Cardenolides have a five-member lactone ring whereas bufadienolides have a six-member lactone ring. The cardenolides have been determined to have a predilection for the α2 and α3 isoforms of Na^+^/K^+^ ATPase, whereas the bufodienolides act primarily on the α1 isoform [28]. Recent studies [9,10,29] demonstrated that CTSs induced the apoptotic signaling and caused cell cycle arrest in CTBs. These findings indicate that CTSs are not only interacting through the Na^+^/K^+^ ATPase, they also enter into the cells via other receptor/receptors and cause the intercellular signaling. The receptor/receptors of CTSs on CTB cell surface has/have not been yet been studied. The aim of the study was to investigate the effects of PNTTU on CTB cell proliferation and expression of some markers of angiogenesis and angiotensin II effect.

## 2. Experimental Section

### 2.1. General Information

The CTB cell culture media DMEM/F-12 was purchased from Invitrogen, Grand Island, NY, USA and cell were incubated in an Isotemp CO_2_ Incubator, Fisher, Waltham, MA, USA. RPMI Media and gels were purchased from Invitrogen, Grand Island, NY, USA. Cell viability and cell proliferation assay kits were purchased from Promega, Madison, WI, USA. Cell migration assay kit was purchased from Cell Biolabs, San Diego, CA, USA. BCA protein assay kit and chemiluminescent substrate were from Pierce, Rockford, IL, USA. The Qunatikine ELISA was purchased from R&D Systems, Minneapolis, MN, USA. The nitrocellulose membranes were from Bio-Rad, Hercules, CA, USA. The primary and secondary antibodies were purchased from Santa Cruz Biotechnology, Paso Robles, CA, USA; Abcam, Cambridge, MA, USA and Jackson ImmunoResearch Laboratories, West Grove, PA, USA. Absorbance was measured on a plate reader, SPECTRAmax 340PC384, Molecular Devices, Sunnyvale, CA, USA. The fluorescence was measured at on a fluorescence plate reader, CytoFluor Series 4000 Fluorescence Multi-Well Plate Reader, Applied Biosystems, Grand Island, NY, USA. The chemiluminence detection system was used LAS-3000 Imaging System, Fuji Photo Film Co., Ltd., Minato-ku, Tokyo, Japan.

### 2.2. Synthesis of PNTTU

To a solution of 4-nitrophenyl isothiocyanate (1.843 g, 10.23 mmol) in THF (50 mL) was added a solution of tris(2-aminoethyl) amine (520 µL, 3.41 mmol) in THF (50 mL) dropwise at room temperature under constant stirring. The reaction mixture was refluxed at 75 °C for 36 h. Then the mixture was cooled to room temperature and was poured over hexane (30–40 mL), yielding a yellow precipitate. Yield: 2.1 g, 89%. ^1^H-NMR (500 MHz, DMSO-*d_6_*, TSP): *δ* 10.17 (s, 3H, Ar-N*H*), 8.17 (s, 3H, CH_2_N*H*), 8.13 (d, *J* = 9.20 Hz, 6H, Ar*H*), 7.78 (d, *J* = 9.15 Hz, 6H, Ar*H*), 3.66 (t, *J* = 6.10 Hz, 6H, NHC*H*_2_), 2.81 (t, *J* = 6.72 Hz, 6H, NC*H*_2_). ^13^C-NMR (125 MHz, DMSO-*d_6_*,): *δ* 179.82 (*C* = S), 146.14 (Ar-*C*), 141.65 (Ar*C*-NO_2_), 124.52 (Ar-*C*H), 120.25 (Ar-*C*H), 51.56 (NH*C*H_2_), 41.83 (N*C*H_2_). M.P 196 °C. ESI-MS: m/z (%) 686.9 (MH^+^). Anal. Calcd. For C_27_H_30_N_10_O_6_S_3_: C, 47.22; H, 4.40; N, 20.39. Found: C, 47.17; H, 4.36; N, 20.02. IR (KBr): ν_N-H_ 3,445 and 3,276 cm^−1^; ν_C=S_ 1,107 cm^−1^; ν_N=O_ 1,511 and 1,332 cm^−1^.

### 2.3. Cell Culture

The human extravillous cytotrophoblast cell line Sw.71 utilized in these studies was derived from first trimester chorionic villus tissue and was kindly provided by Gil G. Mor at the School of Medicine, Yale University (New Haven, CT, USA). These cells are well characterized and share many characteristics with isolated primary cells, including the expression of cytokeratin-7, HLA class I antigen, HLA-G, BC-1, human chorionic gonadotropin, and human placental lactogen [30,31,32,33]. Sw.71 cells were cultured in DMEM/F-12 (Invitrogen, Grand Island, NY, USA) supplemented with 10% fetal bovine serum, 10 mM Hepes, 0.1 mM MEM non-essential amino acids, 1 mM sodium pyruvate and 100 U/mL penicillin/streptomycin. Cells were incubated at 37°C, 5% CO_2_, and 99% humidity (Isotemp CO_2_ Incubator, Fisher, Waltham, MA, USA). 

### 2.4. Effect of PNTTU on CTB Cells Function

Cell viability was measured using a CellTiter Assay (Promega, Madison, WI, US) and treatments of 0, 0.1, 1, 10, and 100 nM PNTTU for 48 h. After the CellTiter-Blue reagent was added to the cells, the absorbance at 520 nm was measured on a plate reader (SPECTRAmax 340PC384, Molecular Devices, Sunnyvale, CA, USA). Cell proliferation was measured using a CellTiter Assay (Promega, Madison, WI, USA), which is a colorimetric method for determining the number of viable cells. Cells were treated with 0, 0.1, 1, 10, and 100 nM PNTTU for 48 h. After CellTiter 96 was added to the cells, the absorbance at 490 nm was measured on a plate reader (SPECTRAmax 340PC384, Molecular Devices, Sunnyvale, CA, USA). Cell migration was measured using a CytoSelect Assay (Cell Biolabs, San Diego, CA, USA) with treatments of 0, 0.1, 1, 10, and 100 nM PNTTU for 48 h. After the CyQuant GR Dye solution was added to the cells, fluorescence was measured at 480 nm/520 nm on a fluorescence plate reader (CytoFluor Series 4000 Fluorescence Multi-Well Plate Reader, Applied Biosystems, Grand Island, NY, USA). 

### 2.5. ELISA for Angiogenic and Anti-angiogenic Factors

Cells were treated with RPMI Media (Invitrogen) containing 0.1, 1, 10, or 100 nM PNTTU for 48 h. The supernatants of PNTTU treated cells were analyzed using a Qunatikine ELISA (R&D Systems, Minneapolis, MN USA) for concentrations of sVEGF R1/Flt-1 (sFlt-1) and VEGF165. These assays employ the quantitative sandwich enzyme immunoassay technique. Monoclonal antibodies specific for sFlt-1 and VEGF165 were pre-coated onto the microplate. Standards and samples are pipetted into the wells and any sFlt-1 and VEGF165 present is bound by the immobilized antibody. After washing away any unbound substances, an enzyme-linked polyclonal antibody specific for either sFlt-1 or VEGF165 is added to the wells. Following a wash to remove any unbound antibody-enzyme reagent, a substrate solution is added to the wells and a color develops in proportion to the amount of sFlt-1 or VEGF bound in the initial step. The color development is stopped and the intensity of the color is measured using a plate reader (SPECTRAmax 340PC384, Molecular Devices).

### 2.6. Western Blots for VEGFR-1, AT_1_, and AT_2_ Receptors

Cells were treated with RPMI Media (Invitrogen) containing 0.1, 1, 10, or 100 nM PNTTU for 48 h. The cell lysates were utilized to measure VEGFR-1, AT_1_, and AT_2_ receptor expression. Protein concentrations of cell lysates were determined using a BCA Protein Assay Kit (Pierce, Rockford, IL, USA). An equal amount of protein of each sample was run on a NuPAGE Novex 4%–12% Bis-Tris Gel (Invitrogen) and transferred to 0.45 μm nitrocellulose membranes (Bio-Rad, Hercules, CA, USA). Membranes were blocked in 5% milk and incubated with VEGFR-1 (Invitrogen), AT_1_, (Santa Cruz Biotechnology, Paso Robles, CA, USA) and AT_2_ (Abcam, Cambridge, MA, USA) antibodies. After the addition of the corresponding secondary antibody (Jackson ImmunoResearch Laboratories, West Grove, PA, USA), proteins were visualized with SuperSignal West Dura Chemiluminescent Substrate (Pierce) and a chemiluminence detection system (LAS-3000 Imaging System). Densitometry was measured using ImageJ (NIH) and normalized using β-actin.

### 2.7. Statistical Methods

Data are presented as mean ± SEM. Data from PNTTU-treated groups were compared to basal (DMSO)-treated groups using a one-way analysis of variance with Tukey’s *post hoc* test. A *p* value of less than 0.05 was considered significant.

## 3. Results and Discussion

### 3.1. Results

#### 3.1.1. PNTTU Inhibited the CTB Cells Function

**Figure 2:**
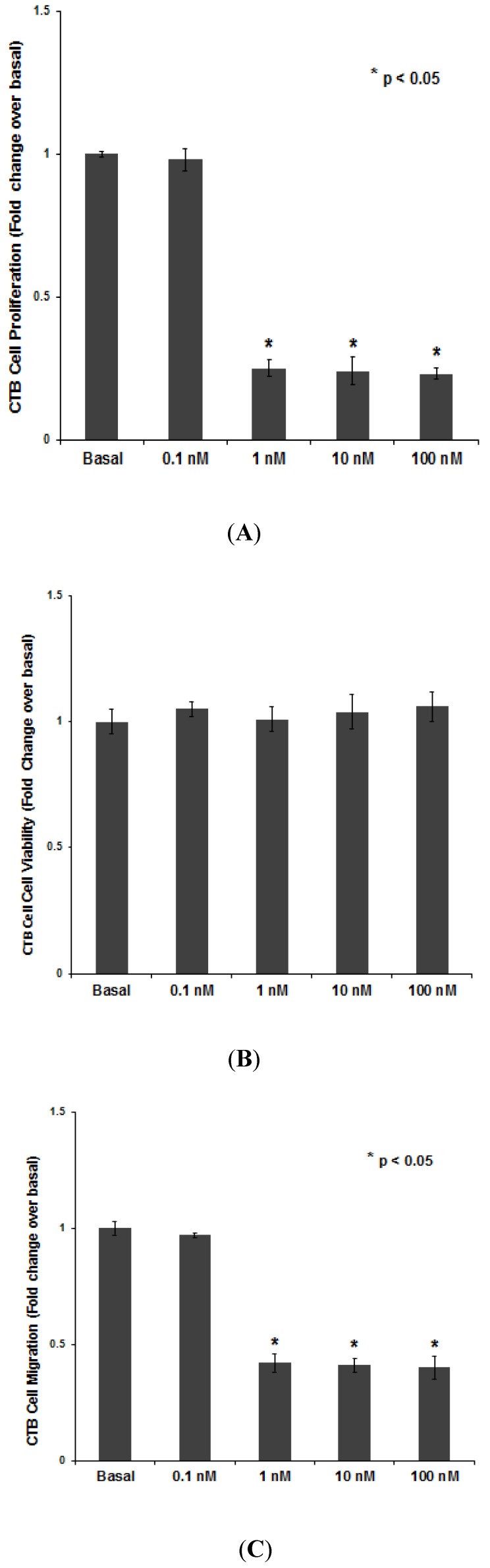
CTB cells were treated with different concentrations of PNTTU and (**A**) Proliferation, (**B**) Cell Viability, and (**C**) Migration were measured. PNTTU significantly (* *p* < 0.05) downregulated the proliferation and migration in CTB cells and had no effect on cell viability. The data are presented as mean ± SEM for four experiments.

CTB cell proliferation was significantly inhibited (approximately 75%) by 1, 10, and 100 nM of PNTTU compared to basal (*p* < 0.05), while 0.1 nM PNTTU had no effect (Figure 2A). These results suggest that PNTTU has an anti-proliferative effect on CTB cell function prior to their differentiation. The anti-proliferative capacity of PNTTU on the cells was not due to a cytotoxic effect of the compound, as evaluated by cell viability (Figure 2B). Cell migration was significantly (*p* < 0.05) inhibited (approximately 58%) by 1, 10, and 100 nM PNTTU when compared to basal, while 0.1 nM PNTTU had no effect (*p* > 0.05) (Figure 2C). 

#### 3.1.2. PNTTU Upregulated Anti-angiogenic Factor (sFlt-1) and Downregulated Angiogenic Factor (VEGF)

**Figure 3:**
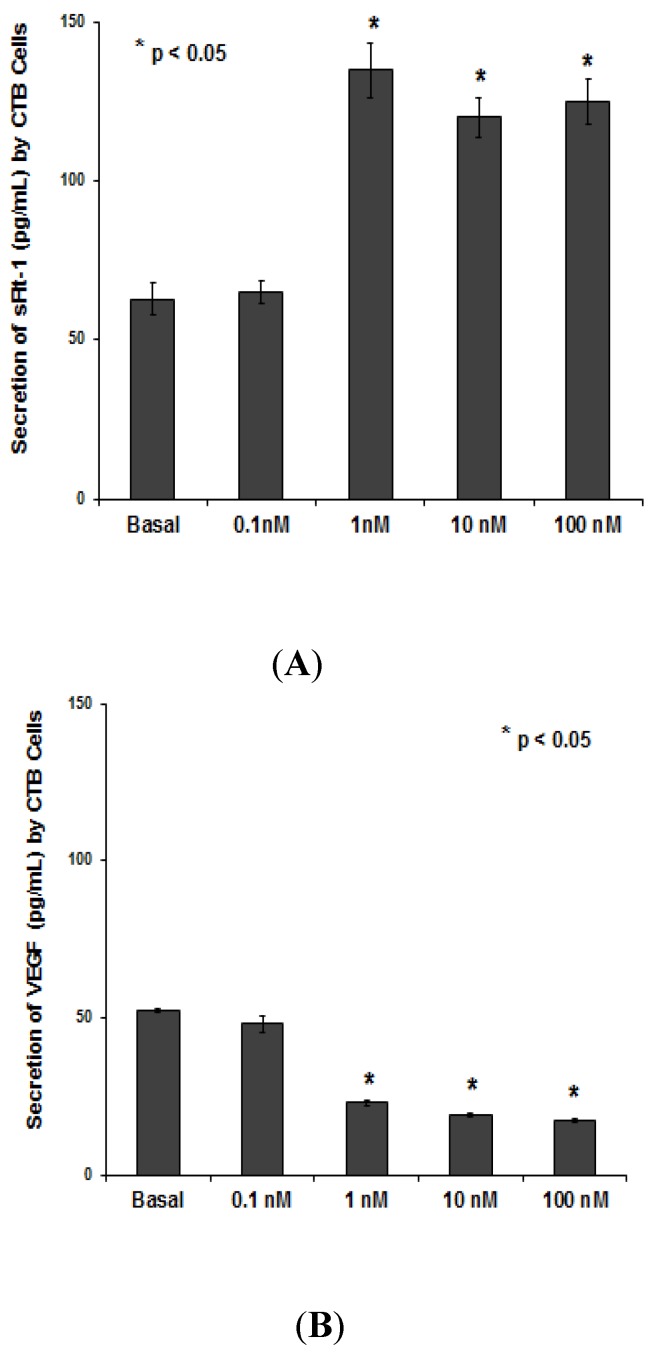
CTB cells were treated with different concentrations of PNTTU and the levels of (**A**) sFlt-1 and (**B**) VEGF were measured in the cell culture media by ELISA. PNTTU significantly (* *p* < 0.05) upregulated the secretion of sFlt-1 and downregulated the secretion of VEGF by CTB cells. The results are presented as the mean ± SEM (n = 6, four replicates each).

The secretion of sFlt-1 was significantly increased in the culture media of CTB cells treated with ≥1 nM by PNTTU (Figure 3A), however, the secretion of VEGF was decreased in the culture media of CTB cells treated with ≥1 nM by PNTTU (Figure 3B).

#### 3.1.3. PNTTU Downregulated VEGFR-1 and AT1 Receptor Expression and Upregulated AT2 Receptor Expression

**Figure 4:**
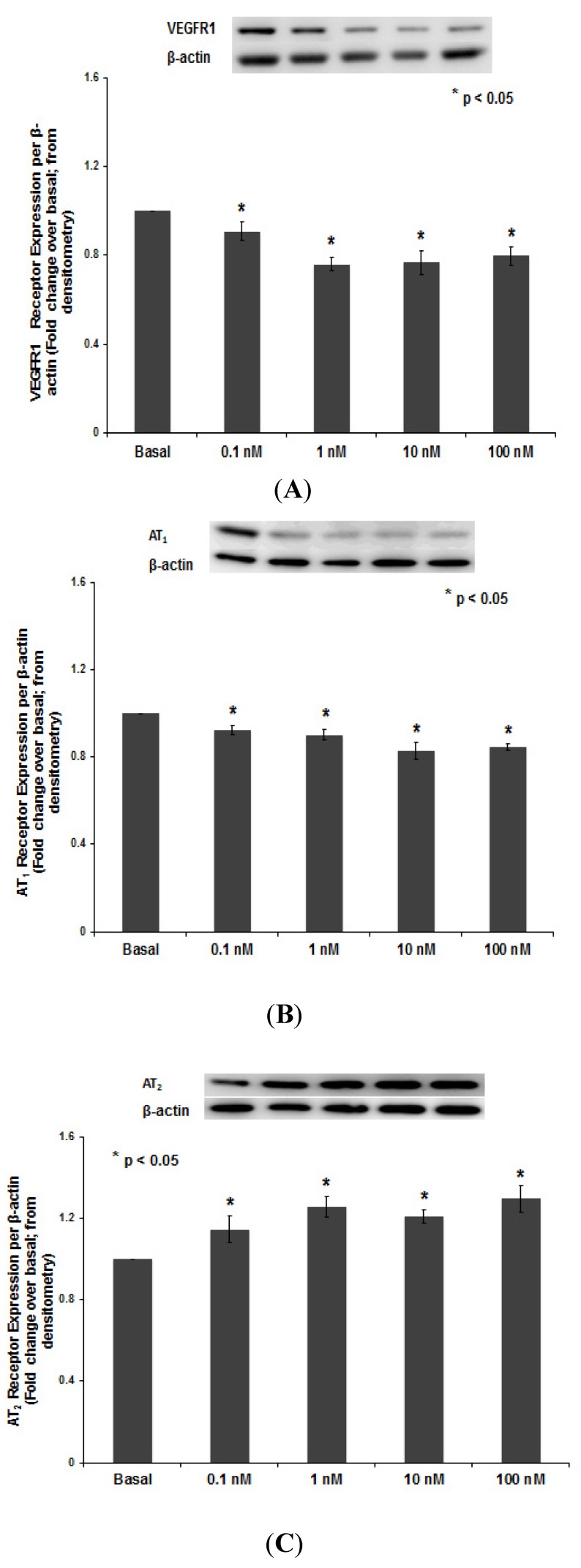
CTB cells were treated with different concentrations of PNTTU and (**A**) VEGFR1, (**B**) AT_1_ receptor, and (**C**) AT_2_ receptors expression were measured in the cell lysates by western Blot. PNTTU significantly (* *p* < 0.05) downregulated the expression of VEGFR1 and AT_1_ receptors in CTB cells and upregulated the expression of AT_2_ receptor. The data are presented as mean ± SEM for four experiments. A blot from a representative experiment is shown in each of the figures.

The VEGFR-1 (Figure 4A) and AT_1_ (Figure 4B) receptors expression was downregulated in ≥1 nM PNTTU treated CTB cells compared to basal, however, the AT_2_ receptor expression was significantly upregulated in ≥1 nM PNTTU treated CTB cells compared to basal (Figure 4C).

### 3.2. Discussion

PNTTU has been shown to inhibit the proliferation and migration of CTB cells. The synthetic receptor is found to down-regulate angiogenic factor (VEGF) and up-regulate anti-angiogenic factor (sFlt-1). Furthermore, PNTTU has shown to upregulate AT_2_ receptor expression and downregulate VEGFR-1 and AT_1_ receptor expression. All these effects of PNTTU are similar with our previous findings of CTSs on CTB cells [8,9,10,34]. Previous study suggested that the PNTTU has strong affinity for an anionic species [18,19,20] particularly for phosphate [19,20]. Therefore, it is suggested that the PNTTU possibly interacts with phosphate groups of Na^+^/K^+^ ATPase, as well as with carboxyl groups of membrane receptors. In the first trimester of pregnancy, the CTB cells of the extravillous trophoblast column migrate through and invade the decidualized endometrium attaching the placenta to the uterus. They then breach and subsequently line uterine blood vessels channeling maternal blood to the rest of the placenta. Adequate CTB invasion sets into motion events that ultimately lead to the remodeling of the maternal vessels which is a crucial phase for proper placental establishment. This process of remodeling is essential for the fetus which needs increased maternal blood flow as the pregnancy progresses. Defects in CTB differentiation are often associated with preE. The amount of interstitial invasion is frequently reduced and the endovascular invasion is consistently rudimentary [35]. Consequently, there is a reduction in uteroplacental perfusion that leads to placental focal ischemia and hypoxia later in pregnancy [36].

Although the exact mechanism of preE continues to elude researchers and clinicians, several theories have been postulated. One theory looks at the roles of bufodeinolides, a subgroup of cardiac steroids (CTSs). The most studied of this group is, MBG, which has been shown to be elevated in both animal models and human subjects with preE in response to increased volume expansion [3,37,38,39]. Others have shown that MBG levels are elevated prior to the onset of hypertension and proteinuria implicating MBG as a factor in the pathogenesis of preE. MBG has also been shown to interfere with proliferation, migration and invasion of CTB cells showing its role in abnormal placentation seen in preE [3,8,9,10]. MBG- and ouabain-induced activation of Jnk, p38, and Src, activation of caspase 9 and 3/7, positive annexin-V staining, and increased secretion of IL-6 in CTB cells suggest that these cardiotonic steroids increase apoptotic signaling and lead to apoptosis in CTB cells [9]. MBG was also shown to upregulate Jnk and p38 phosphorylation and activate apoptosis in rat lung microvascular endothelial (RLME) cells as confirmed by activation of caspases 3/7, 8, and 9 as well as Annexin-V staining [10]. A p38 inhibitor was shown to attenuate the activation of apoptosis by MBG in RLME cells [10]. MBG inhibited proliferation, increased monolayer permeability, decreased ERK1/2 phosphorylation, activated Jnk, p38, and Src phosphorylation, increased the expression of caspase 3/7 indicating the activation of apoptosis in human brain microvascular endothelial (HBME) cells [11].

**Figure 5:**
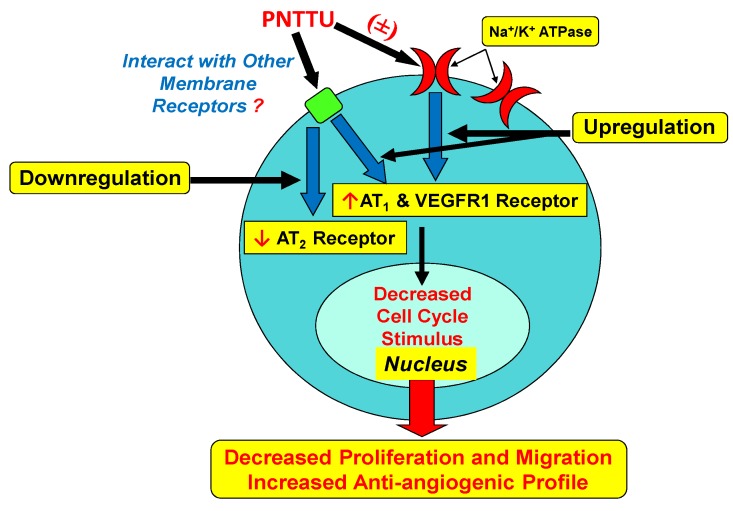
Diagram showing the interactions of the thiourea functional groups in the synthetic receptor PNTTU with membrane receptors via residual carboxyl groups resulting in the decrease of signaling for proliferative and angiogenic profile.

## 4. Conclusions

We propose that the thiourea functional groups in PNTTU interact with membrane receptors possibly through residual carboxyl groups resulting in the decrease of signaling for proliferative and angiogenic profiles (Figure 5). The receptor/ligand binding affinity of PNTTU on CTB cells provides us to design an effective inhibitor to prevent the CTS-induced impairment of CTBs. Research work is undergoing with different synthetic receptors to identify the functional group that is responsible for CTBS dysfunction.

## References

[1] Berg C.J., Atrash H.K., Koonin L.M., Tucker M. (1996). Pregnancy-related mortality in the United States, 1987. Obstet. Gynecol..

[2] Pridjian G., Puschett J.B. (2002). Preeclampsia. Part 1: Clinical and pathophysiologic considerations. Obstet. Gynecol. Surv..

[3] Vu H., Ianosi-Irimie M.R., Pridjian C., Whitbred J.M., Durst J.M., Bagrov A.Y., Fedorova O.V., Pridjian G., Puschett J.B. (2005). The involvement of marinobufagenin in a rat model of human preeclampsia. Amer. J. Nephrol..

[4] Hamlyn J.M., Ringel R., Schaeffer J., Levinson P.D., Hamilton B.P., Kowarski A.A., Blaustein M.P. (1982). A circulating inhibitor of (Na^+^ + K^+^) ATPase associated with essential hypertension. Nature.

[5] Mohammadi K., Liu L., Tian J., Kometiani P., Xie Z., Askari A. (2003). Positive inotropic effect of ouabain on isolated heart is accompanied by activation of signal pathways that link Na^+^/K^+^-ATPase to ERK1/2. J. Cardiovasc. Pharmacol..

[6] Liu J., Tian J., Haas M., Shapiro J.I., Askari A., Xie Z. (2000). Ouabain interaction with cardiac Na^+^/K^+^-ATPase initiates signal cascades independent of changes in intracellular Na^+^ and Ca^2+^ concentrations. J. Biol. Chem..

[7] Iwamoto T. (2006). Vascular Na^+^/Ca^2+^ exchanger: Implications for the pathogenesis and therapy of salt-dependent hypertension. Amer. J. Physiol.-Regul. Integr. C..

[8] Uddin M.N., Horvat D., Glaser S.S., Danchuk S., Mitchell B.M., Sullivan D.E., Morris C.A., Puschett J.B. (2008). Marinobufagenin inhibits proliferation and migration of cytotrophoblast and CHO cells. Placenta.

[9] Uddin M.N., Horvat D., Glaser S.S., Mitchell B.M., Puschett J.B. (2008). Examination of the cellular mechanisms by which marinobufagenin inhibits cytotrophoblast function. J. Biol. Chem..

[10] Uddin M.N., Horvat D., Childs E.W., Puschett J.B. (2009). Marinobufagenin causes endothelial cell monolayer hyperpermeability by altering apoptotic signaling. Amer. J. Physiol.-Regul. Integr. C..

[11] Uddin M.N., Agunanne E., Horvat D., Puschett J.B. (2009). Marinobufagenin causes enhanced permeability in human brain microvascular endothelial cells via apoptotic signaling. J. Am. Soc. Nephrol..

[12] Uddin M.N., Allen S., Jones R., Zawieja D.C., Kuehl T.J. (2012). Pathogenesis of preeclampsia: Marinobufagenin and angiogenic imbalance as biomarkers of the syndrome. Transl. Res..

[13] Ismet B., Emami Khansari M., Pramanik A., Wong B.M., Hossain M.A. (2014). An exclusive fluoride receptor: Fluoride-induced proton transfer to a quinoline-based thiourea. Tetrahedron Lett..

[14] Khansari M.E., Wallace K.D., Hossain M.A. (2014). Synthesis and anion recognition studies of a dipodal thiourea-based sensor for anions. Tetrahedron Lett..

[15] Russ T.H., Pramanik A., Khansari M.E., Wong B.M., Hossain M.A. (2012). A quinoline based bis-urea receptor for anions: A selective receptor for hydrogen sulfate. Nat. Prod. Commun..

[16] Pramanik A., Thompson B., Hayes T., Tucker K., Powell D.R., Bonnesen P.V., Ellis E.D., Lee K.S., Yu H., Hossain M.A. (2011). Seven-coordinate anion complex with a tren-based urea: Binding discrepancy of hydrogen sulfate in solid and solution states. Org. Biomol. Chem..

[17] Busschaert N., Wenzel M., Light M.E., Iglesias-Hernandez P., Perez-Tomas R., Gale P.A. (2011). Structure-activity relationships in tripodal transmembrane anion transporters: The effect of fluorination. J. Am. Chem. Soc..

[18] Werner F., Schneider H.-J. (2000). Complexation of anions including nucleotide anions by open-chain host compounds with amide, urea, and aryl functions. Helv. Chim. Acta.

[19] Dey S.K., Das G. (2011). Encapsulation of trivalent phosphate anion within a rigidified p-stacked dimeric capsular assembly of tripodal receptor. Dalton Trans..

[20] Dey S.K., Das G. (2012). Selective inclusion of PO_4_^3−^ within persistent dimeric capsules of a tris(thiourea) receptor and evidence of cation/solvent sealed unimolecular capsules. Dalton Trans..

[21] Pramanik A., Powell D.R., Wong B.M., Hossain M.A. (2012). Spectroscopic, structural, and theoretical studies of halide complexes with a urea-based tripodal receptor. Inorg. Chem..

[22] Bagrov A.Y., Shapiro J.I., Fedorova O.V. (2009). Endogenous cardiotonic steroids: Physiology, pharmacology, and novel therapeutic targets. Pharmacol. Rev..

[23] Blaustein M.P. (1977). Sodium ions, calcium ions, blood pressure regulation and hypertension: A reasessment and a hypothesis. Amer. J. Physiol..

[24] Dmitrieva R.I., Doris P.A. (2003). Ouabain is a potent promoter of growth and activator of ERK1/2 in ouabain-resistant rat renal epithelial cells. J. Biol. Chem..

[25] Saunders R., Scheiner-Bobis G. (2004). Ouabain stimulates endothelin release and expression in human endothelial cells without inhibiting the sodium pump. Eur. J. Biochem..

[26] Chueh S.C., Guh J.H., Chen J., Lai M.K., Teng C.M. (2001). Dual effects of ouabain on the regulation of proliferation and apoptosis in human prostatic smooth muscle cells. J. Urol..

[27] Huang Y.T., Chueh S.C., Teng C.M., Guh J.H. (2004). Investigation of ouabain-induced anticancer effect in human androgen-independent prostate cancer PC-3 cells. Biochem. Pharmacol..

[28] Schoner W., Scheiner-Bobis G. (2007). Endogenous and exogenous cardiac glycosides: Their roles in hypertension, salt metabolism, and cell growth. Amer. J. Physiol. Cell Physiol..

[29] Uddin M.N., Horvat D., DeMorrow S., Agunanne E., Puschett J.B. (2011). Marinobufagenin is an upstream modulator of Gadd45a stress signaling in preeclampsia. Biochim. Biophys. Acta.

[30] Cartwright J.E., Holden D.P., Whitley G.S. (1999). Hepatocyte growth factor regulates human trophoblast motility and invasion: A role for nitric oxide. Brit. J. Pharmacol..

[31] Cartwright J.E., Kenny L.C., Dash P.R., Crocker I.P., Aplin J.D., Baker P.N., Whitley G.S. (2002). Trophoblast invasion of spiral arteries: A novel *in vitro* model. Placenta.

[32] Choy M.Y., Manyonda M.Y. (1998). The phagocytic activity of human first trimester extravillous trophoblast. Hum. Reprod..

[33] Choy M.Y., Whitley G.S., Manyonda I.T. (2000). Efficient, rapid and reliable establishment of human trophoblast cell lines using poly-L-ornithine. Early Preg..

[34] Ehrig J., Horvat D., Fothergill R.E., Allen S.R., Jones R.O., Zawieja D.C., Kuehl T.J., Uddin M.N. (2013). Cardiotonic steroids induce an anti-angiogenic profile in first trimester cytotrophoblast cells. Amer. J. Obstet Gynecol..

[35] Fisher S.J. (2004). The placental problem: Linking abnormal cytotrophoblast differentiation to the maternal symptoms of preeclampsia. Reprod. Biol. Endocrinol..

[36] Kharfi A., Giguère Y., Sapin V., Massé J., Dastugue B., Forest J.C. (2003). Trophoblastic remodeling in normal and preeclamptic pregnancies: Implication of cytokines. Clin. Biochem..

[37] Cunningham F.G., Leveno K.J., Bloom S.L., Hauth J.C., Rouse D.J., Spong C.Y.  (2010). Implantation, Embryogenesis and Placentation. Williams Obstetrics.

[38] Gonick H.C., Ding Y., Vaziri N.D., Bagrov A.Y., Fedorova O.V. (1998). Simultaneous measurement of marinobufagenin, ouabain, and hypertension-associated protein in various disease states. Clin. Exp. Hypertension.

[39] Puschett J.B., Agunanne E., Uddin M.N. (2010). Emerging role of the bufodienolides in cardiovascular and renal disease. Amer. J. Kidney Dis..

